# Self-Pay Emergency Department Visits by Undocumented Patients After 2018 Public Charge Announcement

**DOI:** 10.1001/jamanetworkopen.2025.55081

**Published:** 2026-01-29

**Authors:** Alein Y. Haro-Ramos, Sarah Axeen, Anna Gorman, Todd Schneberk, Annie Ro

**Affiliations:** 1 Department of Health, Society, and Behavior, Joe C. Wen School of Population & Public Health, University of California, Irvine; 2 Schaeffer Center for Health Policy and Economics, University of Southern California, Los Angeles; 3 Department of Health Services Los Angeles County, Los Angeles, California; 4 Department of Emergency Medicine, University of Southern California, Keck School of Medicine, Los Angeles

## Abstract

**Importance:**

The September 2018 public charge announcement led many undocumented immigrants to avoid public programs over immigration concerns. Whether this extended to Emergency Medicaid and increased self-pay emergency department (ED) visits is unclear.

**Objectives:**

To assess whether the likelihood of self-pay ED visits (visits not covered by any third-party payer because patients lacked or declined insurance coverage) increased among likely undocumented patients vs US-born patients after the public charge announcement.

**Design, Settings, and Participants:**

This retrospective cohort study analyzed ED encounters from major safety-net hospitals in Los Angeles County from May 1, 2017, to December 31, 2019. Adult patient encounters were classified as likely undocumented or US born; likely undocumented encounters were identified based on country of birth and lack of a valid Social Security Number and were further refined with insurance information data. Difference-in-differences and event study models estimated changes in self-pay ED visits after the September 22, 2018, public charge expansion announcement, comparing groups over time to capture differential changes. The study was conducted between September 2024 and August 2025.

**Main Outcomes and Measures:**

The main outcome was the proportion of ED visits that were self-paid. Both difference-in-differences and event study approaches were used.

**Results:**

A total of 375 258 ED patient encounters, including 200 852 (53.5%) US born (mean [SD] age, 41.0 [16.2] years; 120 905 [60.2%] female) and 174 406 (46.5%) likely undocumented (mean [SD] age, 47.4 [13.9] years; 92 802 [53.2%] female), were studied. After the announcement, self-pay status for ED visits increased by 2.10 (95% CI, 1.65-2.47) percentage points among undocumented patient encounters compared with US-born encounters, equivalent to approximately 1755 visits. Event study models found that the increase among likely undocumented patients persisted from November 2018 through August 2019, peaking at 17.8%.

**Conclusions and Relevance:**

In this cohort study of ED encounters, likely undocumented patients had an increase of 2.10 (95% CI, 1.65-2.47) percentage points of self-pay for ED visits compared with US-born patients after the 2018 public charge announcement. This finding suggests how anti-immigrant policies can alter payment patterns, carrying implications for patient financial burden and safety-net hospital fiscal sustainability.

## Key Points

**Question:**

Was the September 2018 public charge announcement associated with changes in self-pay emergency department (ED) visits among likely undocumented patients in Los Angeles County’s safety-net hospitals?

**Findings:**

In this cohort study of 375 258 adult ED encounters, the public charge announcement was associated with a mean 2.10–percentage point increase in self-pay visits among likely undocumented patients (equivalent to 1755 additional visits) compared with US-born patients.

**Meaning:**

These results suggest that undocumented patients are more likely than US-born counterparts to self-pay for their ED care, reflecting a chilling effect of public charge.

## Teaser

This cohort study of adult emergency department encounters evaluates the public charge announcement’s role in payment patterns for emergency department care within the safety-net context.

## Introduction

Immigrants’ access to health care is shaped not only by eligibility restrictions but also by fears that using public programs may jeopardize their immigration prospects. In September 2018, the US Department of Homeland Security announced changes to the public charge rule that would expand the criteria under which immigrants could be denied legal status (ie, lawful permanent residency) for using certain public benefits. The announced proposal initially included Medicaid,^1^ and studies have found that Medicaid enrollment among immigrant populations decreased after its announcement.^2,3,4,5^ However, less is known about how these policies affect payment patterns for emergency department (ED) visits, including shifts toward self-pay.

For undocumented adults, who were ineligible for full-scope Medicaid during the study period, the ED serves as a critical safety net. Visit costs are often covered by Emergency Medicaid,^6^ which represents a small share of total Medicaid spending but disproportionately supports hospitals serving large undocumented populations.^7^ Fear of being labeled a public charge may have led to a decrease in Emergency Medicaid enrollment, prompting some patients to self-pay for their ED care. Self-pay encounters occur when patients do not use any third-party payer, either because they lack insurance or decline available coverage, leaving them fully responsible for costs.^8^ These visits can generate substantial hospital bills, straining patients’ finances and imposing uncompensated care burdens on safety-net hospitals primarily serving uninsured populations.^9^

Although prior studies have shown that sociopolitical threats can influence health care–seeking behavior, leading undocumented patients to delay ED visits due to fears of immigration enforcement,^10,11^ other work has found that, despite these fears, the ED remains a critical point of health care access.^12^ Unlike primary care, where use decreased after the 2016 presidential election, ED use among undocumented patients remained stable, highlighting its unique role as a low-barrier form of care option that enables discrete, episodic visits perceived to reduce immigration-related risks.^12^ Recent California studies indicate that immigrants still continue to experience health care gaps and avoid Medicaid amid shifts in federal immigration policy.^13,14^ Changes in how undocumented patients engage with the ED, such as opting for self-pay over Emergency Medicaid, may reflect an adaptive care-seeking strategy to minimize future immigration consequences while still accessing essential care.

We assess the differential association of the proposed public charge announcement on the likelihood of self-pay ED visits in safety-net settings in Los Angeles, California, comparing changes in likely undocumented patients relative to US-born patients. We hypothesize that after the public charge announcement the probability of self-pay ED visits would increase more among likely undocumented patients than among US-born patients relative to the preannouncement period. To our knowledge, this is the first evaluation of the public charge announcement’s role in payment patterns for ED care within the safety-net context.

## Methods

### Data

This retrospective cohort study included all ED visits to Los Angeles Department of Health Services hospitals from May 1, 2017, to December 31, 2019, using electronic medical records (EMRs) and administrative data. The dataset contained patient identifiers, service location, visit disposition, discharge diagnoses, primary payer, and patient demographics. Visits in which patients left before being seen, before treatment was complete, or against medical advice were excluded due to incomplete clinical or insurance data and may not reflect typical ED use. The study followed the Strengthening the Reporting of Observational Studies in Epidemiology (STROBE) reporting guideline. The University of California, Irvine institutional review board approved the study and waived informed consent owing to the use of existing EMR data. The study was conducted between September 2024 and August 2025.

### Sample Selection

We examined 2 categories of immigration status: US born and likely undocumented. We selected US-born patients as the comparison group because, in theory, they are least likely to be affected by the public charge expansion. We used a logical algorithmic approach to identify the likely undocumented population in ED encounters.^15^ If the country of birth was reported as the US, the encounter was classified as a US-born patient; all others were classified as non–US-born patients. Among the US-born group, we excluded encounters with incomplete Social Security Number (SSN) data (n = 41 825) and encounters with likely misclassification errors (eg, if the primary payer was Emergency Medicaid [n = 1459], which provides emergency coverage to individuals ineligible for Medicaid due to their immigration status).^6^ Our analytic sample included 200 852 US-born patient ED encounters.

Among non–US-born patients, those without a 9-digit SSN were classified as likely undocumented because, generally, only immigrants who are lawfully present and authorized to work in the US possess an SSN.^16^ We refined this classification with insurance status: encounters where the primary payer was full-scope Medicaid (n = 23 933) or Medicare (n = 2055) were excluded because undocumented adults were ineligible for these programs during the study period. These excluded encounters were likely from documented immigrants (ie, lawful permanent residents) who did not provide an SSN during their hospital registration. In California, lawful permanent residents are not subject to a 5-year bar on full-scope Medicaid eligibility.^17^ Our analytic sample included 174 406 likely undocumented patient ED encounters. A flowchart detailing the sample selection process is presented in eFigure 1 in Supplement 1, and a breakdown of primary payer composition by groups is presented in eTable 1 in Supplement 1.

### Variables

#### Exposure

The exposure variables were patient immigration status and the September 2018 announcement of the proposed expansion to the public charge rule. Immigration status was defined as likely undocumented (coded as 1) or US born (coded as 0). The US Department of Homeland Security announced the proposed policy change on September 22, 2018, and we excluded that month to avoid misclassification during the immediate policy shock. The period variable was coded as 1 if the ED visit occurred after the announcement (October 2018 to December 2019) and 0 if the visit occurred before the announcement (May 2017 to August 2018).

#### Outcome

The outcome of interest was whether the primary source of payment for an ED visit was recorded as self-pay in the hospital billing system. Self-pay refers to visits where patients did not use third-party insurance due to lack of coverage, refusal to disclose insurance information, or choosing not to enroll in coverage. This definition is consistent with prior research on ED payment classifications.^18,19^

#### Covariates

Covariates included patients’ age, age squared, preferred language, gender, and race and ethnicity. Race and ethnicity were self-reported by patients at registration and recorded in the EMR. Categories included African American or Black, Asian, Hispanic or Latino, Native Hawaiian or Pacific Islander, White, and other (including multiracial or unspecified). Race and ethnicity were included to describe cohort demographics and to account for potential disparities in health care use. We also included an indicator of whether the ED visit resulted in hospital admission to control for patient severity and hospital fixed effects to control for time-invariant differences in hospital-level practices and patient populations that may influence the likelihood of self-pay designation.

### Statistical Analysis

We estimated the association between the public charge announcement and the likelihood of self-pay ED visits using difference-in-differences and event study approaches. Both quasi-experimental methods allow us to examine changes in outcomes after a policy change among affected subgroups, while adjusting for secular trends in a comparison population.^20^

We used multilevel generalized estimating equations with a binomial distribution, logit link, robust SEs, and an exchangeable correlation structure. Multilevel generalized estimating equation accounts for within-patient correlation across multiple ED visits, estimates average population-level effects, and accommodates unbalanced follow-up by incorporating all encounters regardless of variation in visit frequency or timing. Analyses were conducted at the encounter level. We regressed the outcome on indicators for likely undocumented status (vs US born), the postannouncement period, their interaction (capturing the difference-in-differences estimate), and covariates. The interaction term captured the differential association between the public charge announcement and the likelihood of a self-pay ED visit among likely undocumented encounters compared with US-born encounters.

In the event study, we used monthly indicators from May 2017 to December 2019, with September 2018 as the reference, to capture the monthly association between the public charge announcement and self-pay ED visits across groups. Average marginal effects were used to estimate the probability of a self-pay ED visit for each group, averaged over the preannouncement and postannouncement periods for the difference-in-differences estimate and over the 16 months after the announcement for the event study. To test for differential preannouncement trends, we used eventdd in Stata software with clustered SEs at the patient level. All *P* values were determined using 2-sided tests, and results were deemed statistically significant at *P* < .05. All data were analyzed with Stata, version 14 (StataCorp).

#### Secondary Analyses

Secondary analyses included a subgroup analysis restricted to Latino patient encounters to explore potential spillover effects of the policy among US-born Latino patients.^21^ We anticipated that the differential association of the public charge announcement with the probability of self-pay ED visits would be smaller among likely undocumented than among US-born Latino encounters.

#### Robustness Checks

We conducted several robustness analyses. First, we reestimated difference-in-differences and event study models using a gaussian distribution to check that results were not driven by model specification. Second, we tested robustness to the inclusion of encounters where patients left before treatment or screening. Third, we conducted a placebo test comparing US-born adults 65 years or older with younger counterparts because neither group should have been affected by the policy. Fourth, we used January 1, 2018, as a pseudopolicy date to assess whether similar changes in self-pay visits occurred in the absence of the public charge announcement. Fifth, we used an event study specification to examine potential postannouncement changes in ED visit composition (hospital admissions as a proxy for acuity). Sixth, we replicated analyses among non-Latino patients. Finally, we evaluated pre-post changes in Emergency Medicaid visits as a payer-specific check among likely undocumented encounters.

## Results

We analyzed 375 258 ED encounters, including 200 852 (53.5%) among US-born patients and 174 406 (46.5%) among likely undocumented patients, representing 81 981 and 81 139 unique patients, respectively (Table). More than 40% of patients in each group had encounters in both the prepolicy and postpolicy periods (85 764 [42.7%] US born and 72 030 [41.3%] likely undocumented), reflecting the episodic nature of ED care. Before the announcement, 6254 of 107 824 US-born encounters (5.8%) and 6779 of 89 193 likely undocumented encounters (7.1%) were self-pay; after the announcement, these increased to 6571 of 93 028 (7.1%) and 8606 of 85 213 (10.1%), respectively. Mean (SD) age was lower among US-born encounters (preannouncement: 41.0 [16.2] years; postannouncement: 40.1 [16.0] years) than likely undocumented encounters (preannouncement: 47.4 [13.9] years; postannouncement: 46.5 [13.5] years). In the US-born group, 120 905 (60.2%) were male and 79 931 (39.8%) were female; in the likely undocumented group, 81 594 (46.8%) were male, and 92 082 (53.2%) were female. Among US-born encounters, 69 312 (34.5%) were African American or Black, 3348 (1.7%) Asian, 107 491 (53.5%) Hispanic or Latino, 628 (0.3%) Native Hawaiian or Pacific Islander, 19 047 (9.5%) White, and 917 (0.5%) other; among likely undocumented encounters, 1830 (1.0%) were African American or Black, 5771 (3.3%) Asian, 164 556 (94.4%) Hispanic or Latino, 150 (0.09%) Native Hawaiian or Pacific Islander, 2180 (1.2%) White, and 8 (<0.1%) other. Hospital admission rates were similar (US born: 13 478 [12.5%] before and 12 526 [13.5%] after announcement; likely undocumented: 11 328 [12.7%] before and 11 043 [13.0%] after announcement), and mean (SD) ED visits per patient changed minimally (US born: 6.73 [8.59] vs 6.40 [8.30]; likely undocumented: 4.57 [5.39] vs 4.47 [5.42]). Unadjusted monthly trends in self-pay visits are shown in eFigure 2 in Supplement 1.

**Table.** Characteristics of the Study Population

Characteristic	No. (%) of encounters^a^			
	US born (n = 200 852 encounters) [n = 81 981 unique patients]		Likely undocumented (n = 174 406 encounters) [n = 81 139 unique patients]	
	Period 1 (May 2017-August 2018)	Period 2 (October 2018-December 2019)	Period 1 (May 2017-August 2018)	Period 2 (October 2018-December 2019)
Patients, No.	45 361	36 620	42 978	38 161
Patient encounters, No.	107 824	93 028	89 193	85 213
Self-pay ED visits	6254 (5.8)	6571 (7.1)	6779 (7.1)	8606 (10.1)
Emergency Medicaid visits	NA	NA	60 206 (67.8)	54 017 (63.4)
Year				
2017	54 235 (50.3)	NA	44 061 (49.4)	NA
2018	53 589 (49.7)	25 700 (27.6)	45 132 (50.6)	21 984 (25.8)
2019	NA	67 328 (72.4)	NA	63 229 (74.2)
Age, mean (SD), y	41.0 (16.2)	40.1 (16.0)	47.4 (13.9)	46.5 (13.5)
Hospital				
1	55 206 (51.2)	48 227 (51.8)	44 686 (50.1)	43 892 (51.5)
3	36 445 (33.8)	31 149 (33.5)	18 195 (20.4)	17 442 (20.5)
2	16 174 (15.0)	13 652 (14.7)	26 223 (29.5)	23 879 (28.0)
Gender				
Female	43 022 (39.9)	36 909 (39.7)	47 540 (53.3)	45 262 (53.1)
Male	64 802 (60.1)	56 103 (60.3)	41 653 (46.7)	39 941 (46.9)
Language				
English	100 168 (92.9)	87789 (94.4)	9365 (10.5)	8855 (10.4)
Spanish	5499 (5.1)	4415 (4.7)	76 706 (86.0)	74 059 (86.9)
Other	2156 (2.0)	824 (0.9)	3033 (3.4)	2299 (2.7)
Race and ethnicity				
African American or Black	37 738 (35.0)	31 574 (33.9)	981 (1.1)	849 (1.0)
Asian	1833 (1.7)	1515 (1.6)	3122 (3.5)	2649 (3.1)
Hispanic or Latino	57 039 (52.9)	50 452 (54.2)	84 020 (94.2)	80 536 (94.5)
Native Hawaiian or Pacific Islander	323 (0.3)	305 (0.3)	89 (0.1)	61 (0.1)
White	10 243 (9.5)	8804 (9.5)	1070 (1.2)	1110 (1.3)
Multiracial or unspecified	539 (0.5)	378 (0.4)	NA	8 (<0.1)
Admitted	13 478 (12.5)	12 526 (13.5)	11 328 (12.7)	11 043 (13.0)
No. of ED visits per patient, mean (SD)	6.73 (8.59)	6.40 (8.30)	4.57 (5.39)	4.47 (5.42)

Abbreviations: ED, emergency department; NA, not applicable.

^a^ Unless otherwise indicated.

**Figure 1.**
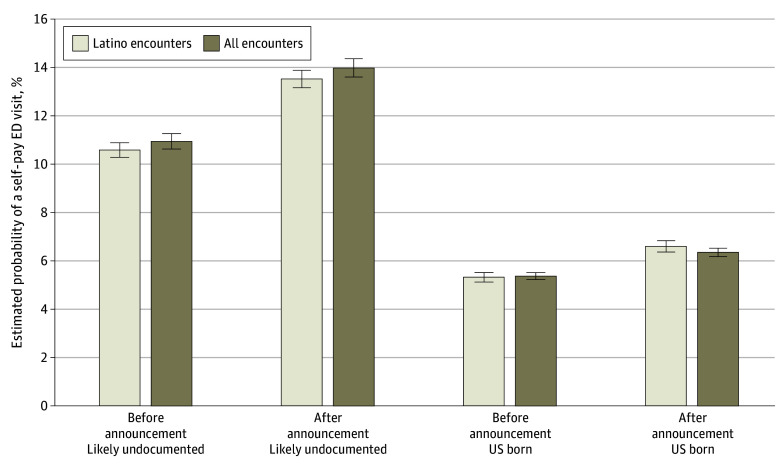
Adjusted Estimated Probability of Self-Pay Emergency Department (ED) Visits Before and After Public Charge Announcement Among Likely Undocumented Patient Encounters Compared With US-Born Patient Encounters The announcement of the public charge expansion was made in September 2018. Error bars indicate SDs.

Results from the difference-in-differences models indicate a greater increase in the likelihood of self-pay ED visits among likely undocumented patients compared with US-born counterparts after the public charge announcement (Figure 1; eTables 2 and 3 in Supplement 1). Based on marginal effects from difference-in-differences estimates, likely undocumented patients had a 2.10–percentage point (95% CI, 1.65-2.47 percentage points) higher probability of a self-pay ED visit after the announcement than US-born patients, corresponding to 1755 (95% CI, 1406-2105) self-pay ED visits among likely undocumented patients. Restricting to Latino encounters, the difference-in-differences estimate was slightly attenuated (1.70 percentage points; 95% CI, 1.21-2.10 percentage points) (eTables 2 and 4 in Supplement 1).

**Figure 2.**
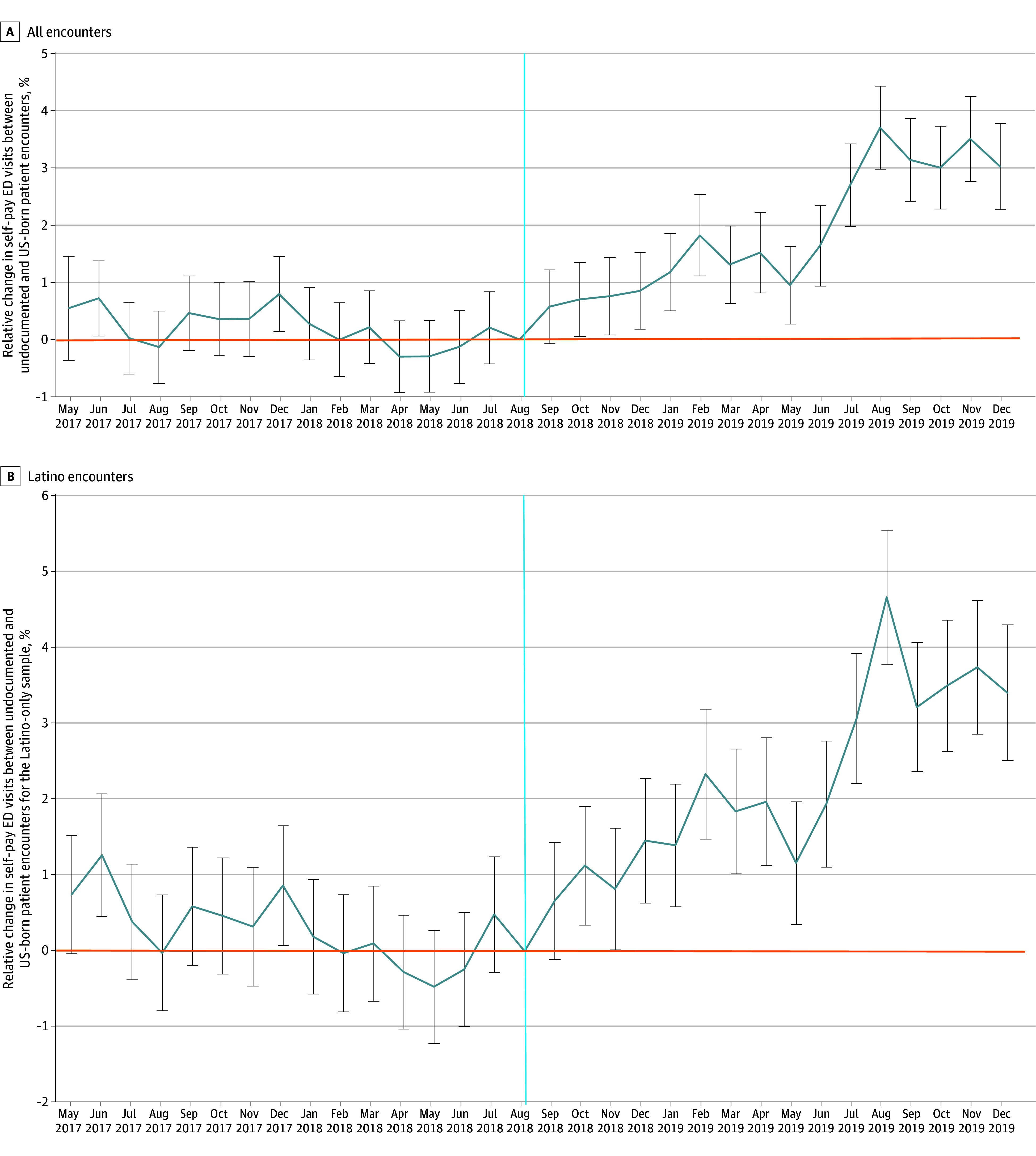
Adjusted Event-Study Estimates of Public Charge Announcement Association on Likelihood of Self-Pay Emergency Department (ED) Visits Among Likely Undocumented and US-Born Patients The figure displays the percentage point difference (95% CI) between likely undocumented and US-born patients in the probability of a self-pay ED visit relative to the reference period (16 months before the public charge announcement, beginning in May 2017). Points to the left of September 2018 (before announcement) represent leads. In contrast, points to the right (after announcement) represent lags, illustrating the event’s immediate and sustained association with likely undocumented patients’ probability of a self-pay ED visit relative to US born. The vertical line represents August 2018, a month before the public charge announcement. There were no significant differential trends in self-pay ED visits before the public charge announcement, which lends support to the event study estimates. Estimates are derived from the event-study model with monthly event-time indicators using eventdd in Stata with clustered SEs (error bars) at the patient level.

Based on the event study, we found no significant differential trends in self-pay ED visits before the public charge announcement, supporting the parallel-trends assumption (Figure 2A). Among all encounters, beginning in November 2018, the relative probability of a self-pay ED visit increased steadily among likely undocumented patient encounters compared with US-born patient encounters, reaching approximately 3.40 percentage points by August 2019 and remaining elevated through year end (Figure 2A). In secondary analyses of the Latino subgroup, preannouncement differences between likely undocumented and US-born encounters were statistically indistinguishable from 0. After the announcement, self-pay ED visits among likely undocumented Latino patients increased more sharply than in the full sample, reaching approximately 4.70 percentage points by mid-2019 and remaining elevated through late 2019, indicating a stronger behavioral response (Figure 2B).

**Figure 3.**
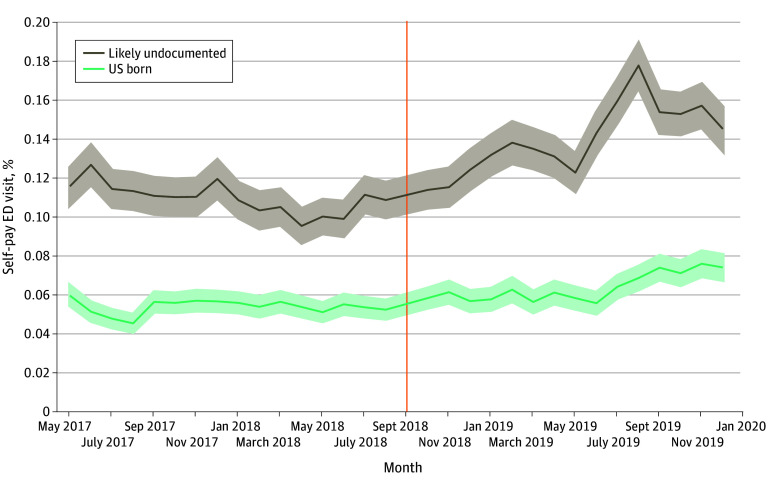
Adjusted Estimated Probabilities of Self-Pay Emergency Department (ED) Visits by Month Relative to the Public Charge Announcement Among Likely Undocumented and US-Born Patient Encounters Estimates are derived from a multilevel generalized estimating equations model with monthly event-time indicators from May 2017 to December 2019. The figure displays the percentage point estimates and 95% CIs (shaded areas) from the mean marginal effects. The vertical line represents September 2018 (the month of the public charge announcement). Points to the right of the orange vertical line (after announcement) illustrate the event’s immediate and sustained association with likely undocumented patients’ probability of a self-pay ED visit relative to US born.

For US-born patient encounters, the probability of a self-pay ED visit remained stable between November 2018 and June 2019 (5.6%-6.2%) (Figure 3; eTables 5 and 6 in Supplement 1), increasing modestly from July 2019 to a peak of 7.6% in November. Likely undocumented patient encounters showed a steady monthly increase beginning in November 2018, peaking in August 2019 at 17.8% (a 6.20–percentage point increase from 11.6%). Increases persisted thereafter, averaging 3 to 4 percentage points above baseline through year end.

### Sensitivity Analyses Results

Results were robust to alternative model specifications (eTable 3 in Supplement 1) and inclusion of previously excluded incomplete ED encounters (eTable 7 in Supplement 1). No differential associations of the public charge announcement on self-pay visits were observed in the placebo test among US-born patients (eTable 8 in Supplement 1). We found no differential change in the likelihood of an ED-originating hospital admission, indicating no substantial change in encounter composition across groups (eTable 9 in Supplement 1). We found decreases in Emergency Medicaid visits among likely undocumented patients after the announcement (−1.75 percentage points; 95% CI, −2.15 to −1.34 percentage points) (eTable 10 in Supplement 1). A placebo analysis using January 1, 2018, as the policy shock showed no differential change in self-pay ED visits between likely undocumented and US-born patients (eTable 11 in Supplement 1).

## Discussion

This study examined the association between the 2018 public charge announcement and self-pay ED visits in safety-net hospitals. After the announcement, likely undocumented patients experienced a significant, sustained increase in self-pay ED visits, beginning soon after the announcement and peaking in August 2019, whereas US-born patients showed smaller, delayed increases beginning in mid-2019.

The increases among likely undocumented patients may reflect decisions to avoid enrollment in public programs due to immigration-related concerns. The mean number of ED visits per patient did not change between the prepolicy and postpolicy periods; rather, approximately 1755 visits shifted to self-pay (visits that Emergency Medicaid may have fully covered). Among likely undocumented patients, the probability of an Emergency Medicaid visit decreased by 1.80 percentage points after the announcement, consistent with the increase in self-pay visits (eTable 10 in Supplement 1).

Based on Los Angeles Department of Health Services charge data, this shift represents approximately $9.95 million in billed charges ($5670 per uncomplicated ED visit). Because charge-master rates exceed reimbursement, the likely revenue loss, based on a mean Medicaid reimbursement of $273 per visit,^22^ is closer to $0.5 million. Patients who choose self-pay are responsible for hospital bills, but payments are limited by their financial capacity and often result in uncompensated care. Such patterns may reduce reimbursement to safety-net hospitals from programs such as Emergency Medicaid and the Global Payment Pool, suggesting that immigration policy shapes payer mix and can disproportionately strain hospitals serving low-income populations.

Although the public charge expansion marked a major policy shift, concurrent immigration enforcement measures may have contributed to observed increases in self-pay ED visits.^23^ These measures included the January 2019 Migrant Protection Protocols (Remain in Mexico),^24^ midyear US Immigration and Customs Enforcement raid announcements in major California cities,^25,26^ and a July 2019 rule accelerating deportations.^27^ Such actions may have heightened fears of immigration consequences, influencing care-seeking and coverage decisions.

We also observed an increase in self-pay ED visits among US-born patients beginning in mid-2019. This trend may partly reflect changes related to the Patient Protection and Affordable Care Act (ACA), including the 2019 repeal of the individual mandate, which led to a national increase in uninsured rates, particularly among the Latino population, and a modest increase in ED visits.^28^ Immigration policy spillover effects may have contributed as well, given that more than half of US-born encounters were among Latino patients. Prior research shows that US-born Latinos with noncitizen family members often avoid public programs due to perceived immigration consequences.^29^ Secondary analysis, restricted to Latino patient encounters, revealed smaller differences between undocumented and US-born patients, consistent with shared behavioral responses to exclusionary policies. In contrast, among patients who are not Latino, there were no differential changes in self-pay ED visits between undocumented and US-born patients until July 2019 (eFigure 3 in Supplement 1). Together, these findings suggest that the mid-2019 increase in self-pay visits among US-born patients likely reflects a combination of spillover effects within Latino mixed-status families and broader coverage instability following ACA changes.

Our estimates are based on clinical encounters in Los Angeles, which has a relatively immigrant-inclusive policy environment and longstanding safety-net programs for undocumented residents. These findings likely represent conservative estimates. In states with less inclusive Medicaid structures or more aggressive enforcement, shifts from Emergency Medicaid to self-pay could be larger. We capture the initial behavioral response to the 2018 public charge announcement, providing a baseline for potential subsequent shifts in payment patterns among likely undocumented patients as immigration policies became more restrictive. Recently, several states have expanded Medicaid to certain undocumented groups, yet some eligible patients may continue to self-pay, potentially increasing uncompensated care in safety-net systems.

### Strengths and Limitations

A key strength of this study is the focus on an undocumented patient population identified through validated methods, offering greater specificity than studies of Latino populations overall. The use of detailed encounter-level data provides objective measures of health care use, overcoming limitations of self-reported data. By finding an association between restrictive immigration policy changes and measurable shifts in care payment patterns, this study contributes evidence on how anti-immigrant policy shapes payment patterns for ED care.

This study also has several limitations. First, as a retrospective observational analysis from a single health system, unmeasured confounding cannot be fully ruled out. Second, undocumented status was inferred indirectly to protect patient privacy, potentially introducing misclassification; however, internal validation analyses (eFigure 1 in Supplement 1) and existing research on the low prevalence of legal nonimmigrants among Latino populations suggest that such misclassification is likely minimal.^30^ Third, we lacked measures of income or educational attainment that may have influenced self-pay status; however, all patients sought care in county hospitals, suggesting similar socioeconomic profiles.

## Conclusions

In this cohort study of ED encounters, self-pay status increased among likely undocumented patient encounters after the 2018 public charge announcement, relative to US-born patient encounters. Although likely undocumented patients were eligible for Emergency Medicaid, some may have self-paid out of concern that enrolling in public programs could jeopardize future immigration prospects. Many patients are likely unable to cover these costs, resulting in uncompensated care for safety-net hospitals. Health systems can reduce the chilling effects of exclusionary immigration policies by protecting patient privacy, partnering with legal organizations to clarify which programs are considered in public charge determinations, and simplifying access to financial assistance programs.

## Notes

Supplement 1. **eTable 1.** Breakdown of groups by primary payer composition, N=375,258 **eTable 2.** Difference-in-Differences of Self-Pay Emergency Department Visits After the Announcement of Public Charge in September 2018, Predicted Probability Estimated Using Average Marginal Effects, Estimated for All Encounters (N=375,258) and Latino Subgroup (N=272,047) **eTable 3.** Difference-In-Differences Estimates of the Association Between the Public Charge Expansion Announcement and Self-Pay Emergency Department Visits, N=375,258 **eTable 4.** Difference-In-Differences Estimates of the Association Between the Public Charge Expansion Announcement and Self-Pay/Uninsured Emergency Department Visits, Los Angeles, California, May 2017 to December 2019, N=272,047, Latino only subsample **eTable 5.** Event Study Estimates of the Association Between the Public Charge Expansion Announcement and Self-Pay/Uninsured Emergency Department Visits, Los Angeles, California, May 2017 to December 2019 (n=375,258) **eTable 6.** Predicted Probabilities of Self-Pay ED Visits from Event Study Model (n=375,258) **eTable 7.** Event Study Estimates of the Association Between the Public Charge Expansion Announcement and Self-Pay/Uninsured Emergency Department Visits, Los Angeles, California, May 2017 to December 2019 (Including Encounters of People Who Left Against Medical Advice, Before Treatment Was Complete, and Before Medical Exam), N=388,328 **eTable 8.** Event Study Estimates of the Association Between the Public Charge Expansion Announcement and Self-Pay Emergency Department Visits, Los Angeles, California, May 2017 to December 2019 (Restricted to US-born Encounters [<65 Vs. 65+]), N=200,852 **eTable 9.** Event Study Estimates of the Association Between the Public Charge Expansion Announcement and Emergency Department Originating Hospital Admission, Los Angeles, California, May 2017 to December 2019 (Assessing Compositional Differences), N=375,258 **eTable 10.** Pre-Post Changes in Emergency Medicaid Coverage Among Likely Undocumented Patient Encounters (n=174,406) **eTable 11.** Difference-In-Differences Estimates of the Association Between the Public Charge Expansion Announcement and Self-Pay Emergency Department Visits Using a Placebo Window (January 2018), N=375,258 **eFigure 1.** Study Selection **eFigure 2.** Descriptive Monthly Time Trends in Emergency Department Visits in Which the Primary Payor Is Self-Pay by Control (US-Born Patient Encounters) and Treatment Groups (Likely Undocumented Patient Encounters) From May 2017 to December 2019 **eFigure 3.** Adjusted Event Study Estimates of Public Charge Announcement Association on Likelihood of Self-Pay ED Visits Among Non-Latino US-Born and Non-Latino Likely Undocumented

Supplement 2.
Data Sharing Statement
