# Supplementary material for: Self-Pay Emergency Department Visits by Undocumented Patients After 2018 Public Charge Announcement
Source: JAMA Netw Open. 2026 Jan 29;9(1):e2555081. doi: 10.1001/jamanetworkopen.2025.55081 (PMC12856681; doi:10.1001/jamanetworkopen.2025.55081)
Supplement: Supplement 1. — eTable 1. Breakdown of groups by primary payer composition, N=375,258 eTable 2. Difference-in-Differences of Self-Pay Emergency Department Visits After the Announcement of Public Charge in September 2018, Predicted Probability Estimated Using Average Marginal Effects, Estimated for All Encounters (N=375,258) and Latino Subgroup (N=272,047) eTable 3. Difference-In-Differences Estimates of the Association Between the Public Charge Expansion Announcement and Self-Pay Emergency Department Visits, N=375,258 eTable 4. Difference-In-Differences Estimates of the Association Between the Public Charge Expansion Announcement and Self-Pay/Uninsured Emergency Department Visits, Los Angeles, California, May 2017 to December 2019, N=272,047, Latino only subsample eTable 5. Event Study Estimates of the Association Between the Public Charge Expansion Announcement and Self-Pay/Uninsured Emergency Department Visits, Los Angeles, California, May 2017 to December 2019 (n=375,258) eTable 6. Predicted Probabilities of Self-Pay ED Visits from Event Study Model (n=375,258) eTable 7. Event Study Estimates of the Association Between the Public Charge Expansion Announcement and Self-Pay/Uninsured Emergency Department Visits, Los Angeles, California, May 2017 to December 2019 (Including Encounters of People Who Left Against Medical Advice, Before Treatment Was Complete, and Before Medical Exam), N=388,328 eTable 8. Event Study Estimates of the Association Between the Public Charge Expansion Announcement and Self-Pay Emergency Department Visits, Los Angeles, California, May 2017 to December 2019 (Restricted to US-born Encounters [<65 Vs. 65+]), N=200,852 eTable 9. Event Study Estimates of the Association Between the Public Charge Expansion Announcement and Emergency Department Originating Hospital Admission, Los Angeles, California, May 2017 to December 2019 (Assessing Compositional Differences), N=375,258 eTable 10. Pre-Post Changes in Emergency Medicaid Coverage Among Likely Undocumented Patie [file jamanetwopen-e2555081-s001.pdf]

dSupplemental Online Content

Haro-Ramos AY, Axeen S, Gorman A, Schneberk T, Ro A. Self-pay emergency department visits by undocumented patients after 2018 public charge announcement. *JAMA Netw Open*. 2026;9(1):e2555081. doi:10.1001/jamanetworkopen.2025.55081

eTable 1. Breakdown of groups by primary payer composition, N=375,258
eTable 2. Difference-in-Differences of Self-Pay Emergency Department Visits After the Announcement of Public Charge in September 2018, Predicted Probability Estimated Using Average Marginal Effects, Estimated for All Encounters (N=375,258) and Latino Subgroup (N=272,047)
eTable 3. Difference-In-Differences Estimates of the Association Between the Public Charge Expansion Announcement and Self-Pay Emergency Department Visits, N=375,258
eTable 4. Difference-In-Differences Estimates of the Association Between the Public Charge Expansion Announcement and Self-Pay/Uninsured Emergency Department Visits, Los Angeles, California, May 2017 to December 2019, N=272,047, Latino only subsample
eTable 5. Event Study Estimates of the Association Between the Public Charge Expansion Announcement and Self-Pay/Uninsured Emergency Department Visits, Los Angeles, California, May 2017 to December 2019 (n=375,258)
eTable 6. Predicted Probabilities of Self-Pay ED Visits from Event Study Model (n=375,258)
eTable 7. Event Study Estimates of the Association Between the Public Charge Expansion Announcement and Self-Pay/Uninsured Emergency Department Visits, Los Angeles, California, May 2017 to December 2019 (Including Encounters of People Who Left Against Medical Advice, Before Treatment Was Complete, and Before Medical Exam), N=388,328
eTable 8. Event Study Estimates of the Association Between the Public Charge Expansion Announcement and Self-Pay Emergency Department Visits, Los Angeles, California, May 2017 to December 2019 (Restricted to US-born Encounters [<65 Vs. 65+]), N=200,852
eTable 9. Event Study Estimates of the Association Between the Public Charge Expansion Announcement and Emergency Department Originating Hospital Admission, Los Angeles, California, May 2017 to December 2019 (Assessing Compositional Differences), N=375,258
eTable 10. Pre-Post Changes in Emergency Medicaid Coverage Among Likely Undocumented Patient Encounters (n=174,406)
eTable 11. Difference-In-Differences Estimates of the Association Between the Public Charge Expansion Announcement and Self-Pay Emergency Department Visits Using a Placebo Window (January 2018), N=375,258
eFigure 1. Study Selection
eFigure 2. Descriptive Monthly Time Trends in Emergency Department Visits in Which the Primary Payor Is Self-Pay by Control (US-Born Patient Encounters) and Treatment Groups (Likely Undocumented Patient Encounters) From May 2017 to December 2019
eFigure 3. Adjusted Event Study Estimates of Public Charge Announcement Association on Likelihood of Self-Pay ED Visits Among Non-Latino US-Born and Non-Latino Likely Undocumented

This supplemental material has been provided by the authors to give readers additional information about their work.

© 2026 Haro-Ramos AY et al. *JAMA Network Open.*

eTable 1. Breakdown of groups by primary payer composition (n=375,258)

Primary Payor	Likely undocumented encounters (n=174,406)	US-born encounters (n=200,852)
Self-pay	8.78%	6.40%
Restricted/Emergency Medi-Cal	65.61%	0.00%
Sheriff	0.49%	5.22%
Hospital Presumptive Eligibility	16.15%	7.25%
Commercial	2.18%	4.68%
Other County Program	6.79%	4.46%
Full-scope Medi-Cal	0.00%	59.24%
Medicare	0.00%	12.75%

© 2026 Haro-Ramos AY et al. *JAMA Network Open.*

eTable 2. Difference-in-Differences Estimates in Self-Pay Emergency Department Visits Among Likely undocumented and US-born patient Encounters Before and After the Announcement of Public Charge in September 2018[a]

		Self-pay ED visits, % (95% Confidence Intervals) Period 1[b]	Self-pay ED visits, % (95% Confidence Intervals) Period 2[b]	Adjusted % Change (95% Confidence Intervals), percentage points
All (n=375,258)	Likely undocumented	10.96 (10.64, 11.28)	14.0 (13.62, 14.38)	+3.04 (2.69, 3.40)
	US-born	5.38 (5.24, 5.53)	6.36 (6.19, 6.54)	+0.98 (.78, .01)
	Diff-in-Diff			2.06 (1.65, 2.47)
Latinos (n=272,047)	Likely undocumented	10.60 (10.3, 10.9)	13.54 (13.18, 13.9)	+2.94 (2.59, 3.3)
	US-born	5.33 (5.13, 5.52)	6.61 (6.38, 6.85)	+1.29 (1.0, 1.54)
	Diff-in-Diff			1.66 (1.21, 2.1)

[a] The estimates represent changes among Likely undocumented patient encounters compared to US-born patient encounters. As secondary analysis, we restricted the sample to all Latino patient encounters. The adjusted models account to age, age squared, gender, hospital fixed effects, year of the encounter, race-ethnicity (except for the Latino-only analysis), emergency department-originating hospital admission indicator, and patients' preferred language at the encounter.

Predicted Probability Estimated Using Average Marginal Effects

[b] Period 1 indicates May 2017 to August 2018, and Period 2 indicates October 2018 to December 2019.

© 2026 Haro-Ramos AY et al. *JAMA Network Open.*

eTable 3. Difference-In-Differences Estimates of the Association Between the Public Charge Expansion Announcement and Self-Pay Emergency Department Visits, N=375,258

	Model 1	Model 2
	Log odds	Beta Coefficient
	[95% CI]	[95% CI]
Likely undocumented patient encounter * ED encounter is post-public charge announcement	0.109*	0.0207*
	[0.06,0.16]	[0.01, 0.03]
ED encounter is post-public charge announcement (Ref. pre-announcement encounter)	0.181*	0.0113*
	[0.14,0.22]	[0.01, 0.01]
Likely undocumented patient encounter (Ref. US-born patient encounter)	0.790*	0.0677
	[0.74,0.84]	[0.06, 0.07]
Hospital (Ref. Hospital 1)		
Hospital 2	0.264*	0.0200*
	[0.23,0.29]	[0.02, 0.02]
Hospital 3	-0.101*	-0.00767*
	[-0.14,-0.07]	[-0.01, -0.01]
Age	-0.0268*	-0.0027
	[-0.03,-0.02]	[-0.00, -0.00]
Age-squared	0.000128*	0.0000173*
	[0.00,0.00]	[0.00, 0.00]
Male (Ref. Female)	0.452*	0.0343*
	[0.42,0.48]	[0.03, 0.04]
Language (Ref. English)		
Spanish	-0.470*	-0.0451*
	[-0.52,-0.42]	[-0.05, -0.04]
Other	-0.0294	-0.00657+
	[-0.11,0.06]	[-0.01, 0.00]
Race/ethnicity (Ref. Hispanic/Latino)		
NH-White	0.0296	0.00205
	[-0.03,0.09]	[-0.00, 0.01]
NH-Asian	0.0448	0.00187
	[-0.04,0.13]	[-0.00, 0.01]
NH-Black	-0.273*	-0.0168*
	[-0.32,-0.23]	[-0.02, -0.01]
NH-NHPI	0.284*	0.0257*
	[0.03,0.54]	[0.00, 0.05]
NH-Other	-0.496*	-0.024
	[-0.87,-0.13]	[-0.04, -0.00]
Treat-and-release encounter (Ref. Hospital admission)	1.385*	0.0588*
	[1.32,1.45]	[0.06, 0.06]

© 2026 Haro-Ramos AY et al. *JAMA Network Open.*

eTable 3. Difference-In-Differences Estimates of the Association Between the Public Charge Expansion Announcement and Self-Pay Emergency Department Visits, N=375,258

Constant	-3.393*	0.0730*
	[-3.51,-3.27]	[0.07, 0.08]

Note: 95% Confidence Intervals in brackets. + p<0.10, * p<0.05. ED=Emergency Department, NH=non-Hispanic, NHPI=Native Hawaiian/Pacific Islander.

Results yielded from population-averaged multilevel model using generalized estimating equations with a binomial distribution and a logit link function. The outcome is regressed on an indicator of whether the ED visit was for an individual who was likely Likely undocumented (vs. US-born), an indicator of whether the visit fell in the pre-announcement period (May 2017– August 2018) or the post-announcement period (October 2018 – December 2019), a multiplicative interaction term between these two indicators, and covariates. In all analyses, we used September 2018 as the reference category. The interaction in the difference-in-difference model captures the differential effect of the public charge announcement on the likelihood of self-pay ED visit among Likely undocumented patients compared to US-born patients. Model 1 was estimated using log-binomial distribution, and Model 2 using Gaussian distribution.

© 2026 Haro-Ramos AY et al. *JAMA Network Open.*

eTable 4. Difference-In-Differences Estimates of the Association Between the Public Charge Expansion Announcement and Self-Pay/Uninsured Emergency Department Visits, Los Angeles, California, May 2017 to December 2019, N=272,047, Latino only subsample

	Model 1	Model 2
	Log odds<br>[95% CI]	Beta Coefficient<br>[95% CI]
Likely undocumented patient encounter * ED encounter is post-public charge announcement	0.235*	0.0167*
	[0.190.28]	[0.01,0.02]
ED encounter is post-public charge announcement (Ref. pre-announcement encounter)	0.0541+	0.00666*
	[0.00, 0.11]	[0.00,0.01]
Likely undocumented patient encounter (Ref. US-born patient encounter)	0.764*	0.0663*
	[0.7, 0.82]	[0.06,0.07]
Hospital (Ref. Hospital 1)		
Hospital 2	0.234*	0.0194*
	[0.20, 0.27]	[0.02,0.02]
Hospital 3	-0.110*	-0.00810*
	[-0.15, -0.07]	[-0.01,-0.01]
Age	-0.0362*	-0.00345*
	[-0.04, -0.03]	[-0.00,-0.00]
Age-squared	0.000253*	0.0000262*
	[0.00, 0.00]	[0.00,0.00]
Male (Ref. Female)	0.522*	0.0419*
	[0.49, 0.55]	[0.04,0.04]
Language (Ref. English)		
Spanish	-0.442*	-0.0414*
	[-0.49, -0.39]	[-0.05,-0.04]
Other	-0.267*	-0.0235*
	[-0.40, -0.13]	[-0.03,-0.01]
	[-0.87,-0.13]	
Treat-and-release encounter (Ref. Hospital admission)	1.422*	0.0653*
	[1.351.50]	[0.06,0.07]
Constant	-3.316*	0.0756*
	[-3.45-3.18]	[0.07,0.08]

Note: 95% Confidence Intervals in brackets. + p<0.10, * p<0.05. ED=Emergency Department

Results yielded from population-averaged multilevel model using generalized estimating equations with a binomial distribution and a logit link function. The outcome is regressed on an indicator of whether the ED visit was for an individual who was likely undocumented (vs. US-born), an indicator of whether the visit fell in the pre-announcement period (May 2017– August 2018) or the post-announcement period (October 2018 – December 2019), a multiplicative interaction term between these two indicators, and covariates. In all analyses, we used September 2018 as the reference category. The interaction in the difference-in-difference model captures the differential effect of the public charge announcement on the likelihood of self-pay ED visit among likely undocumented patients

© 2026 Haro-Ramos AY et al. *JAMA Network Open.*

eTable 4. Difference-In-Differences Estimates of the Association Between the Public Charge Expansion Announcement and Self-Pay/Uninsured Emergency Department Visits, Los Angeles, California, May 2017 to December 2019, N=272,047, Latino only subsample

compared to US-born patients. Model 1 used a log-binomial distribution and Model 2 a Gaussian distribution.

eTable 5. Event Study Estimates of the Association Between the Public Charge Expansion Announcement and Self-Pay/Uninsured Emergency Department Visits, Los Angeles, California, May 2017 to December 2019 (n=375,258)

	Model 1	Model 2
	Log odds	Coefficient
	[95% CI]	[95% CI]
Likely undocumented patient encounter * May 2017	0.0442	0.0025
	[-0.14,0.23]	[-0.01,0.02]
Likely undocumented patient encounter * June 2017	0.312*	0.0203*
	[0.12,0.50]	[0.01,0.03]
Likely undocumented patient encounter * July. 2017	0.268*	0.0146*
	[0.08,0.46]	[0.00,0.03]
Likely undocumented patient encounter * Aug. 2017	0.312*	0.0171*
	[0.12,0.50]	[0.00,0.03]
Likely undocumented patient encounter * Sept. 2017	0.0556	0.00276
	[-0.13,0.24]	[-0.01,0.02]
Likely undocumented patient encounter * Oct. 2017	0.0585	0.00289
	[-0.13,0.24]	[-0.01,0.02]
Likely undocumented patient encounter * Nov. 2017	0.0359	0.00122
	[-0.15,0.23]	[-0.01,0.01]
Likely undocumented patient encounter * Dec. 2017	0.138	0.00916
	[-0.05,0.33]	[-0.00,0.02]
Likely undocumented patient encounter * Jan. 2018	0.0406	0.00148
	[-0.15,0.23]	[-0.01,0.01]
Likely undocumented patient encounter * Feb. 2018	0.0257	-0.000395
	[-0.17,0.22]	[-0.01,0.01]
Likely undocumented patient encounter * March 2018	-0.0111	-0.00214
	[-0.20,0.18]	[-0.02,0.01]
Likely undocumented patient encounter * April 2018	-0.0676	-0.00614
	[-0.26,0.12]	[-0.02,0.01]
Likely undocumented patient encounter * May 2018	0.045	0.00114
	[-0.14,0.23]	[-0.01,0.01]
Likely undocumented patient encounter * June 2018	-0.053	-0.00454
	[-0.24,0.14]	[-0.02,0.01]
Likely undocumented patient encounter * July 2018	0.116	0.00672
	[-0.07,0.30]	[-0.01,0.02]
Likely undocumented patient encounter * Aug. 2018	0.112	0.00589
	[-0.07,0.30]	[-0.01,0.02]
Likely undocumented patient encounter * Sept. 2018	Reference	Reference
Likely undocumented patient encounter * Oct. 2018	0.0111	0.00112

© 2026 Haro-Ramos AY et al. *JAMA Network Open.*

	[-0.17,0.16]	[-0.01,0.01]
Likely undocumented patient encounter * Nov. 2018	0.0161	0.00117
	[-0.17,0.20]	[-0.01,0.01]
Likely undocumented patient encounter * Dec. 2018	0.178+	0.0124*
	[-0.01,0.37]	[0.00,0.03]
Likely undocumented patient encounter * Jan. 2019	0.234*	0.0175*
	[0.05,0.42]	[0.00,0.03]
Likely undocumented patient encounter * Feb. 2019	0.201*	0.0172*
	[0.01,0.39]	[0.00,0.03]
Likely undocumented patient encounter * March 2019	0.284*	0.0214*
	[0.10,0.47]	[0.01,0.03]
Likely undocumented patient encounter * April 2019	0.165+	0.0144*
	[-0.02,0.35]	[0.00,0.03]
Likely undocumented patient encounter * May 2019	0.138	0.0104
	[-0.05,0.33]	[-0.00,0.02]
Likely undocumented patient encounter * June 2019	0.370*	0.0294*
	[0.18,0.56]	[0.02,0.04]
Likely undocumented patient encounter * July 2019	0.351*	0.0328*
	[0.17,0.53]	[0.02,0.05]
Likely undocumented patient encounter * Aug. 2019	0.412*	0.0418*
	[0.23,0.59]	[0.03,0.06]
Likely undocumented patient encounter * Sept. 2019	0.152+	0.0164*
	[-0.03,0.33]	[0.00,0.03]
Likely undocumented patient encounter * Oct. 2019	0.188*	0.0193*
	[0.01,0.37]	[0.01,0.03]
Likely undocumented patient encounter * Nov. 2019	0.147	0.0165*
	[-0.03,0.33]	[0.00,0.03]
Likely undocumented patient encounter * Dec. 2019	0.0771	0.00921
	[-0.11,0.26]	[-0.00,0.02]
Constant	-3.276*	0.0801*
	[-3.43,-3.13]	[0.07,0.09]
95% Confidence Intervals in brackets. + p<0.10, * p<0.05		

Note: ED=Emergency Department, NH=non-Hispanic, NHPI=Native Hawaiian/Pacific Islander

Covariates include age, age squared, self-reported race/ethnicity, self-reported gender, preferred language, an indicator of whether the ED visit resulted in a hospital admission, hospital fixed effects, and monthly fixed effects. Model 1 used a log-link distribution and Model 2 a Gaussian distribution

© 2026 Haro-Ramos AY et al. *JAMA Network Open.*

eTable 6. Predicted Probabilities of Self-Pay ED Visits from Event Study Model (n=375,258)

	US-BORN			LIKELY UNDOCUMENTED		
	Predicted Probability	95% Conf. Intervals		Predicted Probability	95% Conf. Intervals	
May-17	5.97%	5.45%	6.48%	11.58%	10.62%	12.54%
Jun-17	5.14%	4.66%	5.63%	12.68%	11.66%	13.69%
Jul-17	4.78%	4.32%	5.24%	11.44%	10.50%	12.38%
Aug-17	4.54%	4.09%	4.99%	11.33%	10.40%	12.27%
Sep-17	5.64%	5.13%	6.15%	11.08%	10.15%	12.02%
Oct-17	5.59%	5.10%	6.09%	11.02%	10.10%	11.94%
Nov-17	5.70%	5.18%	6.22%	11.03%	10.09%	11.98%
Dec-17	5.67%	5.16%	6.18%	11.95%	10.97%	12.94%
Jan-18	5.59%	5.09%	6.09%	10.86%	9.96%	11.76%
Feb-18	5.39%	4.88%	5.91%	10.34%	9.40%	11.28%
Mar-18	5.65%	5.14%	6.16%	10.51%	9.59%	11.43%
Apr-18	5.38%	4.89%	5.88%	9.54%	8.66%	10.42%
May-18	5.11%	4.64%	5.59%	10.02%	9.15%	10.90%
Jun-18	5.52%	5.01%	6.03%	9.90%	9.00%	10.80%
Jul-18	5.37%	4.88%	5.85%	11.14%	10.23%	12.05%
Aug-18	5.25%	4.77%	5.72%	10.87%	9.97%	11.77%
Post September 2018 announcement						
Oct-18	5.87%	5.72%	6.02%	11.32%	10.32%	12.30%
Nov-18	6.08%	5.53%	6.63%	11.54%	10.57%	12.52%
Dec-18	5.67%	5.14%	6.20%	12.42%	11.42%	13.43%
Jan-19	5.75%	5.20%	6.30%	13.19%	12.18%	14.21%
Feb-19	6.24%	5.64%	6.85%	13.82%	12.75%	14.90%
Mar-19	5.64%	5.09%	6.18%	13.50%	12.49%	14.52%
Apr-19	6.10%	5.53%	6.67%	13.13%	12.11%	14.14%
May-19	5.82%	5.26%	6.37%	12.30%	11.33%	13.27%
Jun-19	5.57%	5.02%	6.11%	14.34%	13.28%	15.39%
Jul-19	6.40%	5.84%	6.97%	15.97%	14.87%	17.08%
Aug-19	6.86%	6.26%	7.45%	17.80%	16.66%	18.93%
Sep-19	7.38%	6.76%	7.99%	15.42%	14.34%	16.51%
Oct-19	7.08%	6.46%	7.70%	15.30%	14.24%	16.35%
Nov-19	7.58%	6.93%	8.23%	15.73%	14.61%	16.84%
Dec-19	7.39%	6.74%	8.04%	14.53%	13.41%	15.66%

Predicted probability of self-pay visits among US-born and likely undocumented patients after the announcement of public charge using = average marginal effects (covariates at observed values) based on model prediction for interpretation.

© 2026 Haro-Ramos AY et al. *JAMA Network Open.*

eTable 7. Event Study Estimates of the Association Between the Public Charge Expansion Announcement and Self-Pay/Uninsured Emergency Department Visits, Los Angeles, California, May 2017 to December 2019 (Including Encounters of People Who Left Against Medical Advice, Before Treatment Was Complete, and Before Medical Exam), N=388,328

	Log odds
	[95% CI]
Likely undocumented patient encounter * May 2017	0.00233
	[-0.01,0.02]
Likely undocumented patient encounter * June 2017	0.0205*
	[0.01,0.03]
Likely undocumented patient encounter * July. 2017	0.0145*
	[0.00,0.03]
Likely undocumented patient encounter * Aug. 2017	0.0171*
	[0.00,0.03]
Likely undocumented patient encounter * Sept. 2017	0.0027
	[-0.01,0.02]
Likely undocumented patient encounter * Oct. 2017	0.00261
	[-0.01,0.02]
Likely undocumented patient encounter * Nov. 2017	0.00118
	[-0.01,0.01]
Likely undocumented patient encounter * Dec. 2017	0.00934
	[-0.00,0.02]
Likely undocumented patient encounter * Jan. 2018	0.00125
	[-0.01,0.01]
Likely undocumented patient encounter * Feb. 2018	-0.000538
	[-0.01,0.01]
Likely undocumented patient encounter * March 2018	-0.0021
	[-0.02,0.01]
Likely undocumented patient encounter * April 2018	-0.00599
	[-0.02,0.01]
Likely undocumented patient encounter * May 2018	0.00148
	[-0.01,0.01]
Likely undocumented patient encounter * June 2018	-0.00445
	[-0.02,0.01]
Likely undocumented patient encounter * July 2018	0.00675
	[-0.01,0.02]
Likely undocumented patient encounter * Aug. 2018	0.00593
	[-0.01,0.02]
Likely undocumented patient encounter * Sept. 2018	Reference
Likely undocumented patient encounter * Oct. 2018	0.00013
	[-0.01,0.01]
Likely undocumented patient encounter * Nov. 2018	0.00136
	[-0.01,0.01]
Likely undocumented patient encounter * Dec. 2018	0.0125+

© 2026 Haro-Ramos AY et al. *JAMA Network Open.*

eTable 7. Event Study Estimates of the Association Between the Public Charge Expansion Announcement and Self-Pay/Uninsured Emergency Department Visits, Los Angeles, California, May 2017 to December 2019 (Including Encounters of People Who Left Against Medical Advice, Before Treatment Was Complete, and Before Medical Exam), N=388,328

	[-0.00,0.03]
Likely undocumented patient encounter * Jan. 2019	0.0179*
	[0.00,0.03]
Likely undocumented patient encounter * Feb. 2019	0.0178*
	[0.00,0.03]
Likely undocumented patient encounter * March 2019	0.0252*
	[0.01,0.04]
Likely undocumented patient encounter * April 2019	0.0149*
	[0.00,0.03]
Likely undocumented patient encounter * May 2019	0.0157*
	[0.00,0.03]
Likely undocumented patient encounter * June 2019	0.0306*
	[0.02,0.04]
Likely undocumented patient encounter * July 2019	0.0359*
	[0.02,0.05]
Likely undocumented patient encounter * Aug. 2019	0.0455*
	[0.03,0.06]
Likely undocumented patient encounter * Sept. 2019	0.0203*
	[0.01,0.03]
Likely undocumented patient encounter * Oct. 2019	0.0259*
	[0.01,0.04]
Likely undocumented patient encounter * Nov. 2019	0.0155*
	[0.00,0.03]
Likely undocumented patient encounter * Dec. 2019	0.0119+
	[-0.00,0.03]
Constant	0.0803*
	[0.07,0.09]

95% confidence intervals in brackets, + p<0.10, * p<0.05
Note: Covariates include age, age squared, self-reported race/ethnicity, self-reported gender, preferred language, an indicator of whether the ED visit resulted in a hospital admission, hospital fixed effects, and monthly fixed effects.

© 2026 Haro-Ramos AY et al. *JAMA Network Open.*

eTable 8. Event Study Estimates of the Association Between the Public Charge Expansion Announcement and Self-Pay/Uninsured Emergency Department Visits, Los Angeles, California, May 2017 to December 2019 (Restricted to US-born Encounters [<65 Vs. 65+]), N=200,852

	log odds
	95% CI
US-born (65+) patient encounter * May 2017	0.0359
	[-0.68,0.75]
US-born (65+) patient encounter * June 2017	0.456
	[-0.27,1.18]
US-born (65+) patient encounter * July. 2017	-0.261
	[-1.00,0.48]
US-born (65+) patient encounter * Aug. 2017	0.177
	[-0.57,0.92]
US-born (65+) patient encounter * Sept. 2017	0.0115
	[-0.73,0.75]
US-born (65+) patient encounter * Oct. 2017	0.00612
	[-0.75,0.76]
US-born (65+) patient encounter * Nov. 2017	0.630+
	[-0.04,1.30]
US-born (65+) patient encounter * Dec. 2017	0.302
	[-0.40,1.01]
US-born (65+) patient encounter * Jan. 2018	-0.0465
	[-0.76,0.66]
US-born (65+) patient encounter * Feb. 2018	0.296
	[-0.41,1.01]
US-born (65+) patient encounter * March 2018	0.159
	[-0.54,0.85]
US-born (65+) patient encounter * April 2018	0.182
	[-0.52,0.89]
US-born (65+) patient encounter * May 2018	0.513
	[-0.17,1.19]
US-born (65+) patient encounter * June 2018	0.276
	[-0.44,0.99]
US-born (65+) patient encounter * July 2018	-0.352
	[-1.12,0.42]
US-born (65+) patient encounter * Aug. 2018	0.394
	[-0.28,1.07]
US-born (65+) patient encounter * Sept. 2018	Reference
Likely undocumented patient encounter * Oct. 2018	-0.209
	[-1.00,0.34]
US-born (65+) patient encounter * Nov. 2018	-0.289
	[-1.10,0.53]
US-born (65+) patient encounter * Dec. 2018	-0.506
	[-1.35,0.34]
US-born (65+) patient encounter * Jan. 2019	-0.621
	[-1.59,0.34]

© 2026 Haro-Ramos AY et al. *JAMA Network Open.*

eTable 8. Event Study Estimates of the Association Between the Public Charge Expansion Announcement and Self-Pay/Uninsured Emergency Department Visits, Los Angeles, California, May 2017 to December 2019 (Restricted to US-born Encounters [<65 Vs. 65+]), N=200,852

US-born (65+) patient encounter * Feb. 2019	0.0929
	[-0.75,0.94]
US-born (65+) patient encounter * March 2019	-0.0475
	[-0.90,0.80]
US-born (65+) patient encounter * April 2019	0.368
	[-0.37,1.10]
US-born (65+) patient encounter * May 2019	0.316
	[-0.46,1.09]
US-born (65+) patient encounter * June 2019	0.18
	[-0.65,1.01]
US-born (65+) patient encounter * July 2019	0.275
	[-0.50,1.05]
US-born (65+) patient encounter * Aug. 2019	-0.748
	[-1.80,0.31]
US-born (65+) patient encounter * Sept. 2019	-0.343
	[-1.12,0.44]
US-born (65+) patient encounter * Oct. 2019	-0.474
	[-1.36,0.41]
US-born (65+) patient encounter * Nov. 2019	0.281
	[-0.44,1.00]
US-born (65+) patient encounter * Dec. 2019	0.464
	[-0.24,1.17]
Constant	-4.695
	[-4.94,-4.45]

95% confidence intervals in brackets, + p<0.10, * p<0.05
Note: Covariates include age, age squared, self-reported race/ethnicity, self-reported gender, preferred language, an indicator of whether the ED visit resulted in a hospital admission, hospital fixed effects, and monthly fixed effects.

© 2026 Haro-Ramos AY et al. *JAMA Network Open.*

eTable 9. Event Study Estimates of the Association Between the Public Charge Expansion Announcement and Emergency Department Originating Hospital Admission, Los Angeles, California, May 2017 to December 2019 (Assessing Compositional Differences), N=375,258

	Log odds
	[95% CI]
Likely undocumented patient encounter * May 2017	0.157*
	[0.00628,0.308]
Likely undocumented patient encounter * June 2017	0.132+
	[-0.0228,0.286]
Likely undocumented patient encounter * July. 2017	0.0523
	[-0.0965,0.201]
Likely undocumented patient encounter * Aug. 2017	0.168*
	[0.0178,0.318]
Likely undocumented patient encounter * Sept. 2017	0.0832
	[-0.0697,0.236]
Likely undocumented patient encounter * Oct. 2017	0.213*
	[0.0636,0.362]
Likely undocumented patient encounter * Nov. 2017	-0.0401
	[-0.194,0.114]
Likely undocumented patient encounter * Dec. 2017	0.117
	[-0.0347,0.269]
Likely undocumented patient encounter * Jan. 2018	-0.0207
	[-0.171,0.129]
Likely undocumented patient encounter * Feb. 2018	0.0501
	[-0.104,0.204]
Likely undocumented patient encounter * March 2018	0.0419
	[-0.109,0.192]
Likely undocumented patient encounter * April 2018	0.0194
	[-0.133,0.172]
Likely undocumented patient encounter * May 2018	0.0456
	[-0.105,0.196]
Likely undocumented patient encounter * June 2018	0.133+
	[-0.0185,0.285]
Likely undocumented patient encounter * July 2018	0.059
	[-0.0899,0.208]
Likely undocumented patient encounter * Aug. 2018	0.0179
	[-0.132,0.168]
Likely undocumented patient encounter * Sept. 2018	Reference
	[0,0]
Likely undocumented patient encounter * Oct. 2018	
Likely undocumented patient encounter * Nov. 2018	0.142
	[-0.0120,0.297]
Likely undocumented patient encounter * Dec. 2018	0.0709
	[-0.0825,0.224]
Likely undocumented patient encounter * Jan. 2019	0.038

© 2026 Haro-Ramos AY et al. *JAMA Network Open.*

eTable 9. Event Study Estimates of the Association Between the Public Charge Expansion Announcement and Emergency Department Originating Hospital Admission, Los Angeles, California, May 2017 to December 2019 (Assessing Compositional Differences), N=375,258

	[-0.114,0.190]
Likely undocumented patient encounter * Feb. 2019	-0.00564
	[-0.162,0.151]
Likely undocumented patient encounter * March 2019	-0.0949
	[-0.247,0.0576]
Likely undocumented patient encounter * April 2019	-0.165
	[-0.331,0.0309]
Likely undocumented patient encounter * May 2019	-0.0468
	[-0.199,0.105]
Likely undocumented patient encounter * June 2019	-0.0222
	[-0.177,0.133]
Likely undocumented patient encounter * July 2019	0.0124
	[-0.141,0.165]
Likely undocumented patient encounter * Aug. 2019	0.0246
	[-0.128,0.177]
Likely undocumented patient encounter * Sept. 2019	-0.0155
	[-0.168,0.137]
Likely undocumented patient encounter * Oct. 2019	0.00876
	[-0.145,0.162]
Likely undocumented patient encounter * Nov. 2019	0.0139
	[-0.139,0.167]
Likely undocumented patient encounter * Dec. 2019	0.127
	[-0.0296,0.284]
Constant	-3.080*
	[-3.195,-2.966]

95% Confidence Intervals in brackets. + p<0.10, * p<0.05
Note: Covariates include age, age squared, self-reported race/ethnicity, self-reported gender, preferred language, hospital fixed effects, and monthly fixed effects.

In the pre-announcement period, a few scattered monthly estimates reached statistical significance (e.g., May 2017: 0.16 [95% CI, 0.01 to 0.31]; Aug 2017: 0.17 [95% CI, 0.02 to 0.32]; Oct 2017: 0.21 [95% CI, 0.06 to 0.36]), but these were small in magnitude, inconsistent in direction, and not part of a systematic trend, supporting the plausibility of the parallel trends assumption.

© 2026 Haro-Ramos AY et al. *JAMA Network Open.*

eTable 10. Pre-Post Changes in Emergency Medicaid Coverage Among Likely Undocumented Patient Encounters (n=174,406)

	Coefficient
	[95% CI]
ED encounter is post-public charge announcement (Ref. pre-announcement encounter)	-0.0175*
	[-0.02,-0.01]
Hospital (Ref. Hospital 1)	
Hospital 2	-0.0813*
	[-0.09,-0.07]
Hospital 3	0.00915*
	[0.00,0.02]
Age	0.00847*
	[0.01,0.01]
Age-squared	-0.0000581*
	[-0.00,-0.00]
Male (Ref. Female)	-0.176*
	[-0.18,-0.17]
Language (Ref. English)	
Spanish	0.0958*
	[0.09,0.11]
Other	0.0717*
	[0.05,0.09]
Race/ethnicity (Ref. Hispanic/Latino)	
NH-White	-0.261*
	[-0.29,-0.24]
NH-Asian	-0.209*
	[-0.23,-0.19]
NH-Black	-0.210*
	[-0.24,-0.18]
NH-NHPI	-0.169*
	[-0.27,-0.07]
NH-Other	-0.282*
	[-0.52,-0.05]
Treat-and-release encounter (Ref. Hospital admission)	-0.0375*
	[-0.04,-0.03]
Constant	0.408
	[0.38,0.43]

95% confidence intervals in brackets. + $p<0.10$, * $p<0.05$

Results yielded from population-averaged multilevel model using generalized estimating equations with a Gaussian distribution and identity link function.

© 2026 Haro-Ramos AY et al. *JAMA Network Open.*

eTable 11. Difference-In-Differences Estimates of the Association Between the Public Charge Expansion Announcement and Self-Pay Emergency Department Visits Using a Placebo Window (January 2018), N=375,258

	Model 1
	Log odds
	[95% CI]
Likely undocumented patient encounter * ED encounter is post-public charge announcement	-0.0130
	[-0.06, 0.04]
ED encounter is post-public charge announcement (Ref. pre-announcement encounter)	0.107*
	[0.07,0.15]
Likely undocumented patient encounter (Ref. US-born patient encounter)	0.740*
	[0.68, 0.80]
Hospital (Ref. Hospital 1)	
Hospital 2	0.228*
	[0.20,0.26]
Hospital 3	-0.0965*
	[-0.13,-0.06]
Age	-0.0237*
	[-0.03, -0.02]
Age-squared	0.000109*
	[0.00,0.00]
Male (Ref. Female)	0.407*
	[0.38,0.43]
Language (Ref. English)	
Spanish	-0.388*
	[-0.43, 0.35]
Other	-0.0456
	[-0.12,0.03]
Race/ethnicity (Ref. Hispanic/Latino)	
NH-White	0.0251
	[-0.04, 0.09]
NH-Asian	0.0377
	[-0.03, 0.11]
NH-Black	-0.253*
	[-0.30,-0.21]
NH-NHPI	0.263*
	[0.03,0.50]
NH-Other	-0.496*

© 2026 Haro-Ramos AY et al. *JAMA Network Open.*

	[-0.89,-0.10]
Treat-and-release encounter (Ref. Hospital admission)	1.299*
	[1.25, 1.35]
Constant	-3.444*
	[-3.55, -3.33]
Note: 95% Confidence Intervals in brackets. + p<0.10, * p<0.05. ED=Emergency Department, NH=non-Hispanic, NHPI=Native Hawaiian/Pacific Islander.	

Results yielded from population-averaged multilevel model using generalized estimating equations using Gaussian distribution with link identity. The outcome is regressed on an indicator of whether the ED visit was for an individual who was likely undocumented (vs. US-born), an indicator of whether the visit fell in the placebo pre-announcement period (May 2017– December 2017) or the placebo post-announcement period (February 2018 – December 2019), a multiplicative interaction term between these two indicators, and covariates. In all analyses, we used January 2018 as the reference category as a placebo shock. The interaction in the difference-in-difference model captures the differential effect of the public charge announcement on the likelihood of self-pay ED visit among likely undocumented patients compared to US-born patients.

eFigure 1. Study Selection

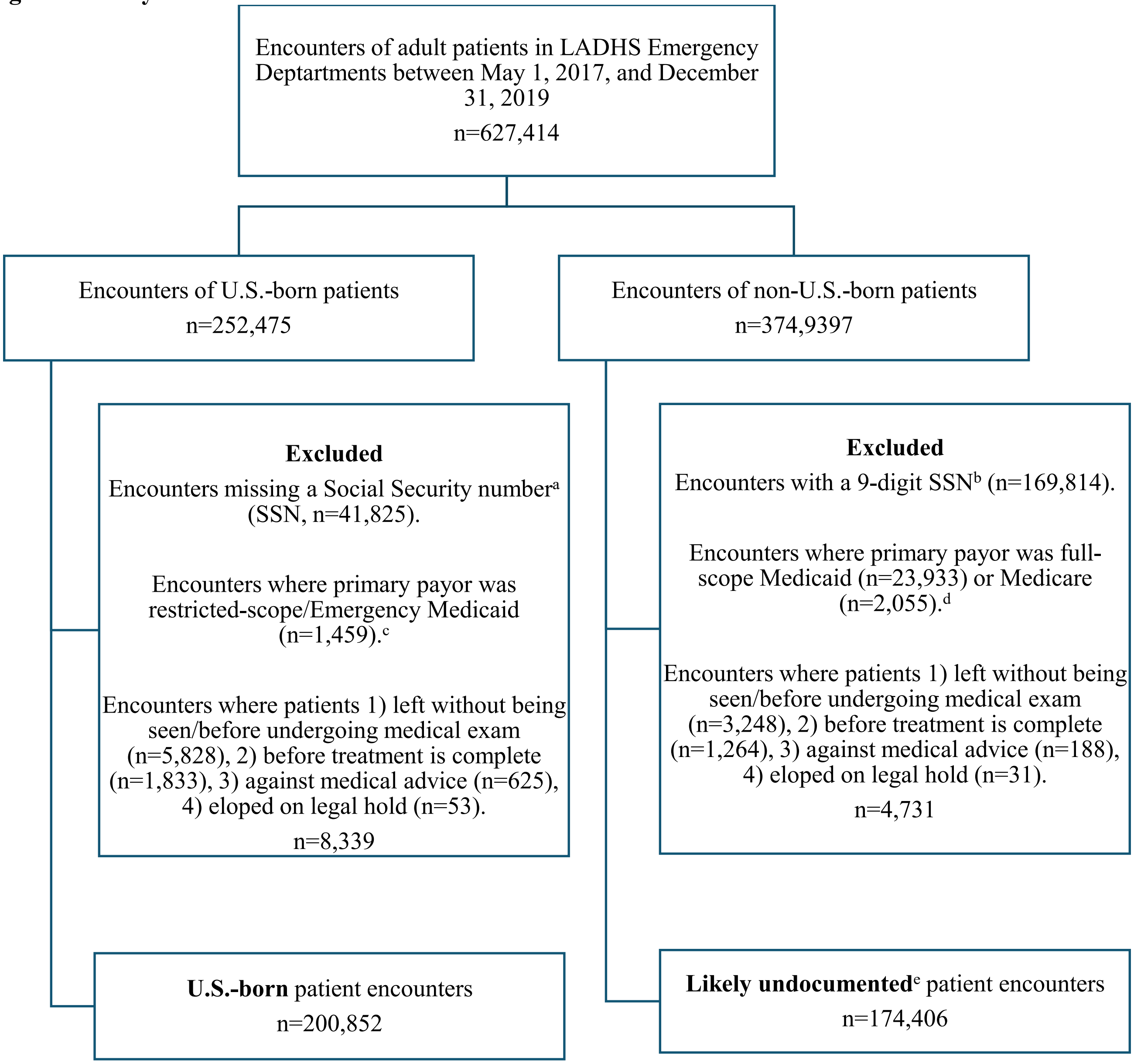

Notes:

a. Due to incomplete data, encounters in which the patient was born in the U.S. and did not provide a 9-digit SSN were excluded from the analyses.
b. Encounters where the patient is not U.S.-born and provides a 9-digit SSN are likely naturalized U.S. citizens or lawful immigrants. It is also possible that this subgroup of immigrant patients

© 2026 Haro-Ramos AY et al. *JAMA Network Open.*

may be undocumented and provided an Individual Taxpayer Identification Number (ITIN) at registration. The IRS issues ITINs to immigrants, regardless of legal status, for tax purposes.

c. These encounters from the US-born control group were excluded because the primary payor was Emergency Medi-Cal, which provides coverage to uninsured individuals who do not qualify for full-scope Medi-Cal due to immigration status. This may be a hospital insurance registration error or an SSN/country of birth reporting error.
d. These likely undocumented patient encounters were excluded because undocumented adults did not qualify for full-scope Medi-Cal or Medicare during the study period; as such, they are likely encounters for lawful immigrants or green card holders who qualified for full-scope Medi-Cal and/or Medicare and did not provide an SSN during their hospital registration.
e. Among the likely undocumented patient encounter group, 31.26% of encounters were enrolled in My Health LA (MHLA) at some point. MHLA was a county-based program offering LADHS healthcare services to likely undocumented people ineligible for full-scope Medi-Cal due to their immigration status. Of these MHLA encounters, the primary payor breakdown is 10.38% HPE, 81.05% restricted/Emergency Medi-Cal, 4.16% self-pay, and 4.41% county program.

© 2026 Haro-Ramos AY et al. *JAMA Network Open.*

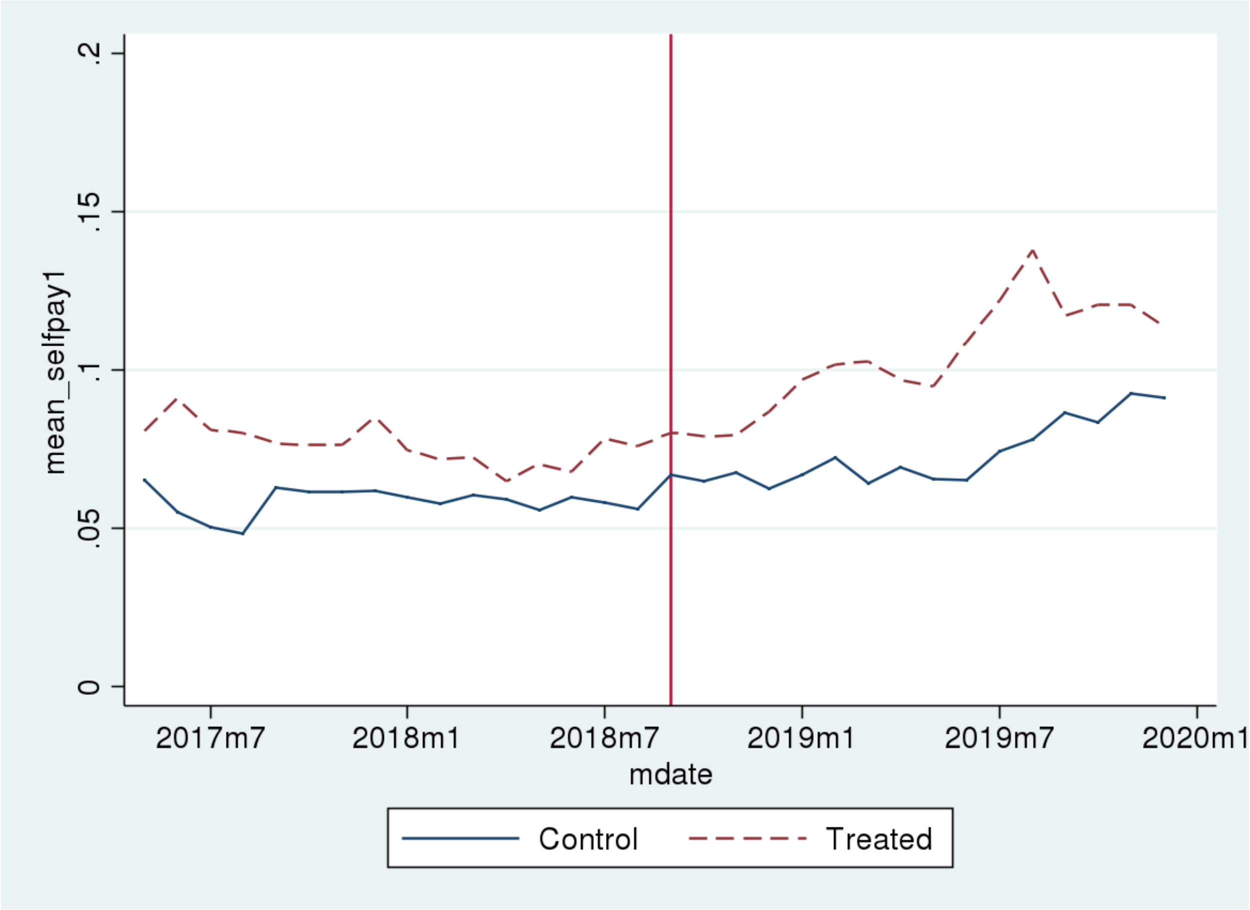

eFigure 2. Unadjusted descriptive monthly time trends in emergency department visits in which the primary payor is self-pay by control (US-born patient encounters) and treatment groups (likely undocumented patient encounters) from May 2017 to December 2019. The vertical line corresponds to September 2018.

© 2026 Haro-Ramos AY et al. *JAMA Network Open.*

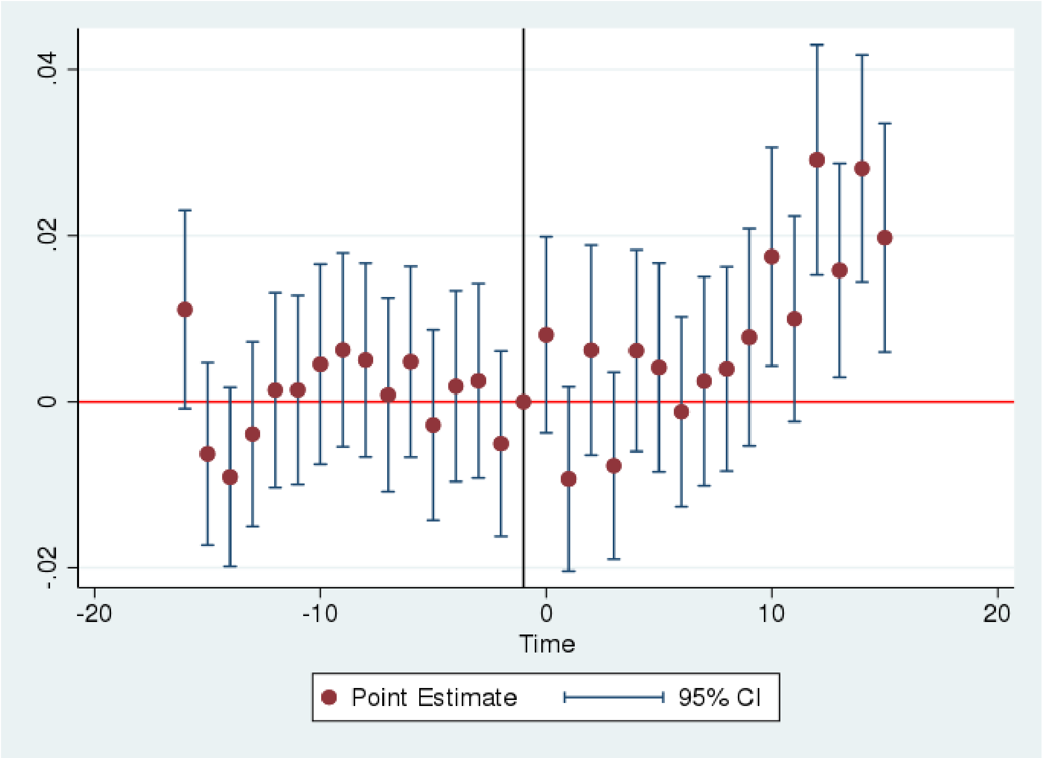

eFigure 3. Adjusted Event Study Estimates of Public Charge Announcement Association on Likelihood of Self-Pay ED Visits Among non-Latino US-born and non-Latino likely undocumented. The figure displays the percentage point difference between non-Latino likely undocumented (n=9,592) and US-born (n=92,384) patient encounters in the probability of a self-pay ED visit relative to the reference period (16 months before the public charge announcement, beginning in May 2017). Confidence intervals at the 95% level are shown for each point estimate. Points to the left of September 2018 (pre-announcement) represent leads. In contrast, points to the right (post-announcement) represent lags, illustrating the event's immediate and sustained association on likely undocumented patients' probability of a self-pay ED visit relative to US-born. The vertical line at -1 represents August 2018, a month before the public charge announcement. There were no significant differential trends in self-pay ED visits before the public charge announcement, which supports the event study estimates. Estimates are derived from the event study model with monthly event-time indicators using *eventdd* in Stata with clustered standard errors at the patient level.

© 2026 Haro-Ramos AY et al. *JAMA Network Open.*
