# Supplementary material for: Self-Pay Emergency Department Visits by Undocumented Patients After 2018 Public Charge Announcement
Source: JAMA Netw Open. 2026 Jan 29;9(1):e2555081. doi: 10.1001/jamanetworkopen.2025.55081 (PMC12856681; doi:10.1001/jamanetworkopen.2025.55081)
Supplement: Supplement 2. — Data Sharing Statement [file jamanetwopen-e2555081-s002.pdf]

Data Sharing Statement

Haro-Ramos. Self-Pay Emergency Department Visits by Undocumented Patients After 2018 Public Charge Announcement. *JAMA Netw Open*. Published January 29, 2026. doi:10.1001/jamanetworkopen.2025.55081

Data
Data available: No

Additional Information
Explanation for why data not available: Data come from patient health records.
